# Unmasking Ehlers-Danlos Syndrome Through Reduction Mammaplasty: An Illustrative Case and a Systematic Literature Review

**DOI:** 10.7759/cureus.113374

**Published:** 2026-07-25

**Authors:** Veronica Gibbons, Vanessa Batubo, Kerri M Woodberry

**Affiliations:** 1 School of Medicine, West Virginia University School of Medicine, Morgantown, USA; 2 School of Medicine, St. George's University, St. George's, GRD; 3 Plastic Surgery, West Virginia University School of Medicine, Morgantown, USA

**Keywords:** breast plastic surgery, breast reduction surgery (brs), ehlers-danlos syndrome, ehlers danlos syndrome hypermobility type (eds-ht), hypermobility disorder

## Abstract

Ehlers-Danlos syndrome (EDS) is characterized by joint hypermobility, skin hyperextensibility, and tissue fragility, which may worsen symptomatic macromastia. Concerns about elevated surgical risk have historically discouraged elective procedures in EDS patients; however, postoperative complications may themselves prompt an EDS diagnosis, as illustrated by our case. This relationship prompted the conduct of a systematic review examining outcomes of reduction mammaplasty in EDS. A 37-year-old woman underwent bilateral inferior pedicle reduction mammaplasty for symptomatic mammary hyperplasia. Persistent intraoperative oozing resulted in postoperative anemia (hemoglobin decline from 13.3 g/dL to 10.4 g/dL). Additional complications included delayed wound healing, hypertrophic scarring, and transient left upper extremity weakness. Hematologic evaluation was unremarkable. Clinical assessment revealed joint hypermobility (Beighton score ≥5/9), positive Walker and Steinberg signs, skin hyperextensibility, easy bruising, and menorrhagia, leading to a clinical diagnosis of hypermobile EDS.

A systematic review of PubMed, Scopus, and Google Scholar (December 1950 to June 2025) was performed and included all study designs. Extracted data included demographics, interventions, complications, revision rates, and satisfaction. Two independent reviewers assessed quality using Joanna Briggs Institute (JBI) critical appraisal tools. Of the 115 identified records, the following four studies met the inclusion criteria: one case report, one case series, one retrospective cohort study, and one clinical reference. All reported cases involved patients with pre-existing EDS diagnoses and demonstrated successful outcomes with symptomatic improvement and few complications. The cohort study consisted of multiple elective procedures, including reduction mammaplasty, and reported fewer complications in EDS patients compared to matched controls, but did not report outcomes specific to the reduction mammaplasty group. The clinical reference emphasized meticulous operative planning. This systematic review with an illustrative case underscores the need for further research and updated clinical guidance. Although existing literature largely challenges the view of EDS as a surgical contraindication, our case demonstrates complications consistent with known EDS-related risks and suggests a potential role for plastic surgeons in identifying undiagnosed EDS.

## Introduction

Ehlers-Danlos syndrome (EDS) encompasses 13 subtypes, as established by the 2017 International Classification, with pathogenic variants identified in 20 genes [1,2]. Hypermobile EDS is the only subtype that currently lacks a confirmed molecular diagnostic test and is diagnosed clinically using the 2017 International Classification, which requires generalized joint hypermobility (evaluated using the Beighton score), additional systemic or musculoskeletal features, and exclusion of other connective tissue disorders [1]. Many EDS subtypes share overlapping clinical features; the two most prevalent forms, classical and hypermobile, are characterized by joint hypermobility and skin fragility with hyperextensibility [1,3,4]. Thus, patients with EDS may be at a greater risk for surgical complications, including suture splitting, wound dehiscence, and delayed healing [3]. Additionally, patients with EDS often present with atrophic scarring and blood vessel fragility, particularly in the vascular subtype, which raises concern for increased bleeding [3,4]. The perceived risk of greater complications has led to a general hesitancy to operate on patients with EDS, particularly in elective surgery [5,6]. However, recent literature has documented favorable outcomes in patients with EDS, prompting re-evaluation of long-standing concerns and reluctance regarding surgery in patients with EDS [7,8].

Reduction mammaplasty is of particular significance to re-evaluate in the context of EDS, as patients with symptomatic macromastia and concomitant EDS may experience exaggerated symptoms. Acute and chronic pain are common manifestations of EDS and may be compounded by macromastia, which independently produces back, neck, and shoulder pain [4,9,10]. Women with EDS often report greater pain intensity and more widespread discomfort than men, particularly in the setting of skin laxity and joint instability [9]. Chronic pain is a major contributor to disability, functional limitation, and psychosocial distress in this population, and reduction mammaplasty has been shown to significantly alleviate symptoms and improve quality of life in patients with macromastia [4,9-12]. Collectively, these findings suggest that patients with coexisting EDS and macromastia may experience greater symptomatic benefit from reduction mammaplasty than the general population.

Likewise, symptoms attributed to macromastia may in some cases be partially explained by an undiagnosed connective tissue disorder. Such a scenario occurred in the illustrative case, in which intraoperative abnormalities ultimately prompted an EDS diagnosis. In contrast to recent reports of favorable outcomes, this patient experienced postoperative complications. To further characterize the outcomes and current clinical recommendations surrounding reduction mammaplasty in individuals with EDS, we performed a systematic literature review. No systematic reviews specifically addressing reduction mammaplasty in this population were identified during the literature search.

## Case presentation

Patient information

A 37-year-old woman (body mass index: 24.54 kg/m², never-smoker, gravida 3 para 2) presented for bilateral reduction mammaplasty for symptomatic mammary hyperplasia with chronic back, neck, and shoulder pain. Prior to surgical consultation, the patient had undergone physical therapy for musculoskeletal pain. She wore a 36G bra cup size, with progressive breast enlargement following two pregnancies. Her past medical history included anxiety, hyperlipidemia, migraine with aura, and postpartum depression. Past surgical history included tubal ligation. Preoperative breast examination revealed sternal notch to nipple distance of 34 cm bilaterally, nipple to nipple distance of 26 cm, nipple to inframammary fold of 19 cm bilaterally, and areolar diameter of 8 cm (right) and 6 cm (left).

Therapeutic intervention

Bilateral reduction mammaplasty was performed using the inferior pedicle technique with a Wise pattern. Specimen weights were 953 g (right) and 1,018 g (left). The operative time was longer than typical breast reductions, spanning just under 5 h. Notably, increased bleeding with persistent oozing was observed from the onset of the procedure and throughout the case. The patient was discharged home in stable condition on the day of surgery.

Follow-up and outcomes

The operation resulted in 300 mL of intraoperative blood loss, causing acute blood loss anemia with a hemoglobin decline from 13.3 g/dL to 10.4 g/dL, meeting the International Society on Thrombosis and Hemostasis Bleeding Assessment Tool criteria for major hemorrhage. Postoperatively, she developed left upper-extremity weakness and sensory loss, though upper-extremity ultrasound was negative for vascular pathology. Additional complications included delayed wound healing and hypertrophic scarring treated with intralesional triamcinolone acetonide injections. Standard hematologic workup was negative; however, the clinical picture raised concern for an underlying connective tissue disorder.

On detailed re-evaluation, the patient disclosed a longstanding history of generalized increased flexibility, chronic back, hip, and rib pain, paresthesia in the left arm and foot, the ability to "tent" the skin on her chest prior to surgery, and unexplained chronic fatigue. The patient reported easy bruising predominantly of the wrists, hands, and thighs, easy oral mucosal bleeding, menorrhagia prior to intrauterine device insertion, and postpartum anemia following both deliveries. Family history was expanded and notable for a maternal grandmother having thyroid disease, notable flexibility, and easy bruising. Physical examination demonstrated bilateral knee hyperextension greater than 10°, passive flexion of both thumbs to the ipsilateral forearm, positive Walker and Steinberg signs bilaterally, and skin hyperextensibility on the dorsum of the hands; the Beighton score was ≥5/9 [1]. Negative genetic workup excluded other types of EDS, so the patient received a clinical diagnosis of hypermobile EDS within a few months of her surgery date. Following the reduction mammaplasty, her back pain continued.

## Discussion

This report of a patient who underwent reduction mammaplasty complicated by intraoperative bleeding, delayed wound healing, and keloid scarring contrasts with recent findings, most notably those of the retrospective cohort study by Fodor et al., which suggest that elective breast surgery can be performed in patients with EDS without increased complication rates [7]. Given the particular benefit that patients with EDS may derive from reduction mammaplasty, a more thorough evaluation of the existing literature is warranted. Accordingly, this systematic review aimed to evaluate all published reports of reduction mammaplasty in patients with EDS, providing a comprehensive overview of outcomes, complication rates, and clinical recommendations.

Systematic literature review

Methods

This systematic review followed Preferred Reporting Items for Systematic Reviews and Meta-Analyses (PRISMA) guidelines, and the study protocol was registered with the International Prospective Register of Systematic Reviews (PROSPERO) (#CRD420251075655) [13]. The study included all study types, such as cohort studies, case series, case reports, and surgical guides, to provide a comprehensive understanding of current recommendations and practices regarding EDS and reduction mammaplasty. Reports that did not focus on or mention reduction mammaplasty procedures on patients with EDS were excluded. Only studies published in English were included; therefore, no translation services were required.

We performed a literature search in the PubMed, Scopus, and Google Scholar databases from December 1950 up to June 2025. The search scripts were designed using keywords and Medical Subject Headings (MeSH) terms, such as “Ehlers-Danlos syndrome,” combined with “reduction mammaplasty," “breast reduction," and “aesthetic surgery.” Additionally, the search strategies were refined to include more general terms such as “plastic surgery," alongside Boolean operators (AND, OR) to locate and include all relevant studies (Table 1).

**Table 1:** Systematic review search scripts for each database.

Database	Search script
PubMed	(((((((Ehlers-Danlos [Title/Abstract]) AND breast reduction [Title/Abstract])) OR ((Ehlers-Danlos [Title/Abstract]) AND mammaplasty [Title/Abstract])) OR ((Ehlers-Danlos [Title/Abstract]) AND plastic surg* [Title/Abstract])) OR ((Ehlers-Danlos [Title/Abstract]) AND cosmetic surg* [Title/Abstract])) OR ((Ehlers-Danlos [Title/Abstract]) AND aesthetic surg* [Title/Abstract])) OR ((Ehlers-Danlos [Title/Abstract]) AND breast surg* [Title/Abstract])
Scopus	TITLE-ABS-KEY("Ehlers-Danlos Syndrome" OR "Ehlers-Danlos" OR EDS OR "connective tissue disorder" OR "collagen disorder") AND TITLE-ABS-KEY("breast surgery" OR "plastic surgery" OR "aesthetic surgery" OR "cosmetic surgery" OR "reconstructive surgery" OR mammaplasty OR "breast reconstruction" OR "breast augmentation" OR "breast implant" OR mastectomy) AND TITLE-ABS-KEY("wound healing" OR healing OR "tissue repair" OR recovery OR "surgical wound") AND TITLE-ABS-KEY(complications OR "surgical complications" OR "postoperative complications" OR hematoma OR seroma OR infection OR "adverse outcomes" OR "treatment outcome" OR "clinical outcome" OR prognosis)
Google Scholar	("Ehlers-Danlos" AND ("breast reduction" OR “reduction mammaplasty”))

**Figure 1:**
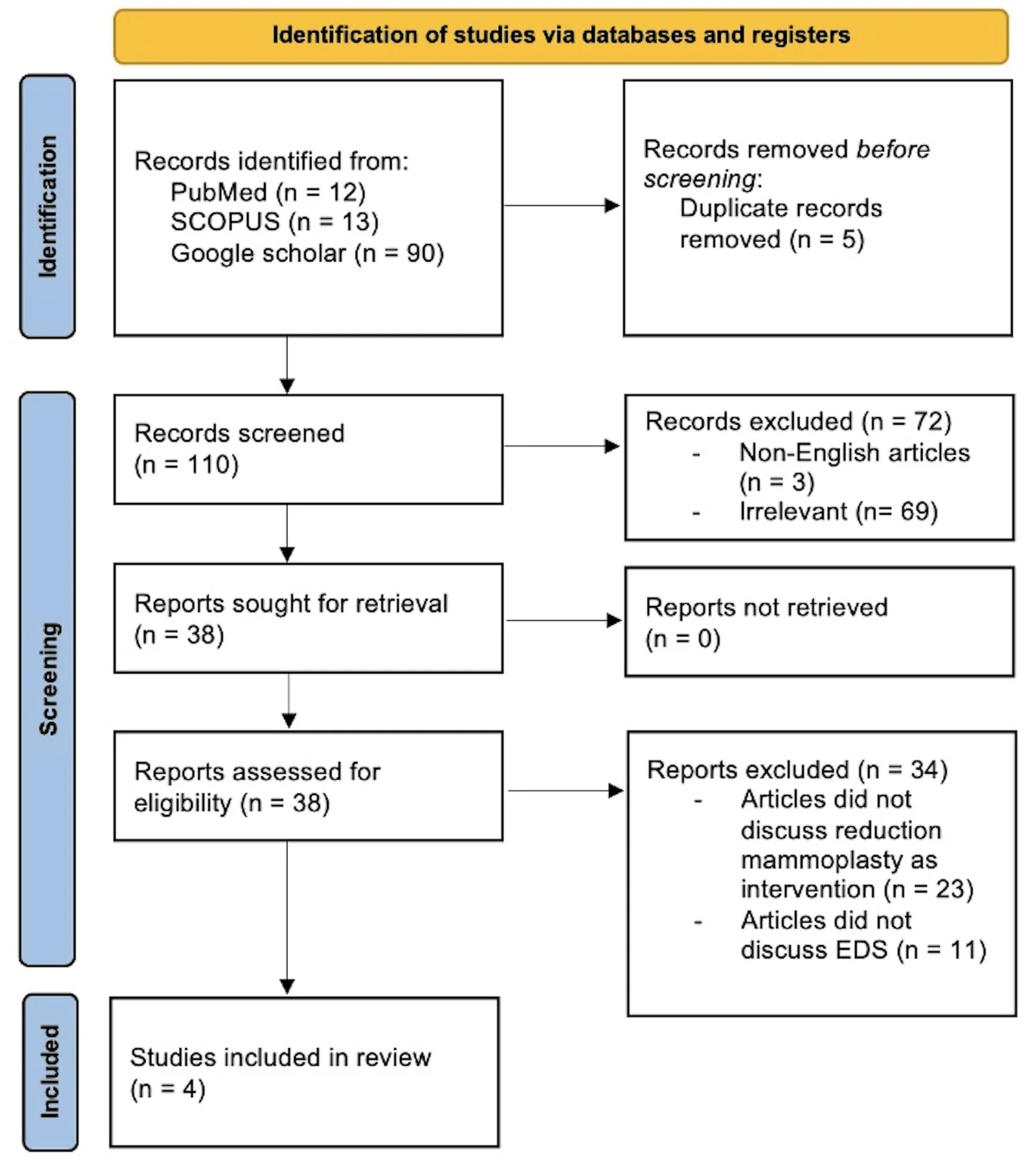
PRISMA flow diagram illustrating the study selection process. PRISMA: Preferred Reporting Items for Systematic Reviews and Meta-Analyses; EDS: Ehlers-Danlos syndrome

The screening and data extraction process was conducted in stages. The first stage consisted of implementing the search strategies and consolidating the identified reports in one location for a cohesive review. Duplicate articles were counted and addressed. Then, in the second stage, the titles and abstracts of the identified studies were assessed against predefined eligibility criteria, and articles that clearly did not meet the standards were excluded. Lastly, full-text versions of the remaining reports were retrieved to continue the assessment (Figure 1). The included reports were thoroughly reviewed for data extraction as follows: author, study design, sample size, demographics, intervention(s), time taken to heal, complications, revision rates, and patient satisfaction. All screening and data collection were conducted independently by two reviewers, with any disagreements resolved through discussion (Table 2). The bias of the included studies was evaluated using the Joanna Briggs Institute (JBI) critical appraisal tools [14-16]. Each study was evaluated against a set of criteria depending on the type of study (Table 3).

**Table 2:** Summary of included studies. *Only 9% of this population underwent reduction mammaplasty. The reported demographics, complication rates, and descriptions represent the entire cohort's elective procedures, not the reduction mammaplasty group specifically. Key findings included study design, demographics of the study population (sex, age), interventions (type of procedures in study population), complications (type and character), total follow-up time, and outcomes including satisfaction and degree of symptom resolution. EDS: Ehlers-Danlos syndrome

Studies	Study design	Sample size	Demographics	Ages (years)	Intervention	Incidence of complications	Type of complication	Total follow-up	Patient satisfaction
Ishimoto et al. (2023) [17]	Case report	1 patient with EDS	Adolescent female	18	Bilateral reduction mammaplasty	None	-	12 months postoperatively	Preoperative symptoms improved (i.e., chronic back, neck, and arm pain)
Morris et al. (2024) [8]	Case series	4 patients with EDS	Adolescent and middle-aged females	18-42	Bilateral reduction mammaplasty in 3 patients, bilateral mastopexy in 1 patient	25% (1 of 4 patients)	Suture splitting in bilateral mastopexy; no complications in bilateral reduction mammaplasty	3-24 months	All patients experienced improvement, if not resolution, of neck, back, and shoulder pain
Fodor et al. (2025) [7]	Retrospective cohort study	100 patients with EDS, 100 matched controls*	EDS: 86% female, matched: 86% female*	EDS: 43.29±17.45; matched: 42.98±18.12*	Elective plastic surgery procedures, including breast reduction (9%), breast augmentation, breast reconstruction, etc.*	Complications were observed in 25% of EDS patients and 35% of matched controls. Minor complications (EDS: 19% vs. controls: 24%), major complications (EDS: 6% vs. controls: 12%)*	The most common among EDS patients was infection. In controls, the most common complication was wound dehiscence. Additional common complications included pathologic scarring, hematoma, and seroma*	EDS median: 115 days, controls median: 93.5 days*	-
Schuurman and Peart (2018) [18]	Clinical guide for EDS	-	-	-	Discusses the concerns for breast reduction, as well as other procedures	States that there is an increased risk of complications, but it can be successful with careful surgical techniques	Risk of poor and disfiguring scars after an operation or injury, and permanent skin pigmentation after hemorrhages. EDS patients are also at greater risk of hemorrhaging and bleeding	-	-

**Table 3:** Summary of bias assessment. Score reports for each included study using JBI critical appraisal tools tailored to the study design and evaluated by two independent reviewers. Studies with ≥80% of criteria met were considered high quality, 60-79% moderate quality, and <60% low quality. JBI: Joanna Briggs Institute

Studies	Bias assessment tool	Score
Ishimoto et al. (2023) [17]	JBI critical appraisal checklist for case reports	7/8
Morris et al. (2024) [8]	JBI critical appraisal checklist for case series	8/10
Fodor et al. (2025) [7]	JBI critical appraisal checklist for cohort studies	9/11
Schuurman and Peart(2018) [18]	JBI critical appraisal checklist for textual evidence: expert opinion	5/6

Summary of Included Studies

The following four studies met the inclusion criteria: one case report, one case series, one retrospective cohort study, and one multidisciplinary clinical reference [7,8,17,18]. Upon review, the included case report appears to overlap with a case from the included case series [17]. One case within the series underwent bilateral mastopexy and experienced suture splitting, whereas all other cases reported uncomplicated reduction mammaplasties without wound-healing issues and showed improvement of macromastia-related symptoms [8]. The retrospective cohort study found that patients with EDS experienced fewer surgical complications (i.e., infection, wound dehiscence, and atrophic scarring) compared with matched controls [7]. The clinical reference discussed surgical considerations, emphasizing the importance of careful operative planning in achieving successful outcomes [18].

Clinical implications

Outcomes

Two of the four included studies provided patient-level EDS-specific reduction-mammaplasty outcomes as follows: a case series by Morris et al. and a case report by Ishimoto et al. detailing four patients with EDS who underwent breast surgery [8,17]. Notably, the case reported by Ishimoto et al. appears to overlap with one of the cases included in Morris et al.'s series, resulting in a total of four individual patient experiences [8,17]. Of the four patients described, three underwent reduction mammaplasty, and one underwent bilateral mastopexy. Both studies reported general satisfaction with surgical outcomes, including significant reductions in pain and improved self-esteem [8,17]. Although some of these patients were managed by a multidisciplinary team comprising rheumatologists, geneticists, physical therapists, and plastic surgeons, the most burdensome symptoms were ultimately resolved through surgical intervention [8,17]. In contrast, the patient discussed in the illustrative case continued to experience back pain alongside other musculoskeletal symptoms following reduction mammaplasty.

Complications

Patients with EDS are at increased risk for surgical complications, including suture splitting, wound dehiscence, delayed healing, atrophic scarring, and excessive bleeding [3,4]. Tissue fragility in classical and hypermobile EDS contributes to abnormal healing and scar formation, while fragile blood vessels and easy bruising are more characteristic of vascular EDS [19]. These risks may deter surgeons from operating on patients with EDS [5,6]. Consistent with these concerns, the patient in the illustrative case experienced anemia, delayed wound healing, and keloid scarring.

Despite these risks, the literature suggests that reduction mammaplasty in EDS patients can be performed with acceptable complication rates. Fodor et al. conducted a retrospective cohort study of 100 EDS patients undergoing plastic surgery, 9% of whom underwent reduction mammaplasty [7]. Although complication rates specific to the reduction mammaplasty subgroup were not reported, the overall EDS cohort had a lower complication rate (25%) compared to matched controls (35%). Similarly, the case series by Morris et al. reported minimal complications as follows: only one of four patients experienced minor issues, and notably, this patient had undergone bilateral mastopexy rather than reduction mammaplasty [8]. The remaining three patients in the series had successful reduction mammaplasties without wound-healing problems or atrophic scarring. While these cases were limited to patients with hypermobile EDS, a subtype generally associated with milder clinical features, this is the most common form of EDS and, therefore, the variant most frequently encountered in clinical practice [7,8].

Recommendations

All studies yielded from this systematic review recommend risk assessment and thorough preoperative planning based on the individual’s symptoms [7,8,17,18]. Similarly, the illustrative case demonstrates an opportunity for identification of patients with possible EDS prior to elective surgery. Physical examination of the patient in their EDS workup demonstrated bilateral knee hyperextension, passive flexion of both thumbs to the ipsilateral forearm, positive Walker and Steinberg signs bilaterally, and skin hyperextensibility on the dorsum of the hands. Plastic surgeons may consider performing at least one of these maneuvers routinely in preoperative visits to identify hypermobile patients (without an EDS diagnosis) who could benefit from more thorough perioperative planning or referral to a rheumatologist.

Likewise, EDS subtype is an important variable that allows for better preoperative planning, as certain EDS subtypes are prone to different complications. However, patients with EDS often do not have a subtype diagnosis [7]. Furthermore, the risks of complications must be weighed on a case-by-case basis for every patient, depending on their EDS symptoms and the predicted impact of the surgical outcomes. Similar recommendations have been proposed for other areas of plastic surgery, including breast reconstruction and microsurgery, but there remains a need for additional literature reevaluating surgical outcomes and recommendations in patients with EDS [19,20].

Limitations

This review is limited by the small number of studies yielded by the systematic literature search, highlighting the need for greater insight into reduction mammaplasty in patients with EDS. The available studies were not only limited in number, but also had small sample sizes. Upon review, the included case report appears to overlap with a case from the included case series, further limiting our results. Likewise, the included study by Fodor et al. did not report complication rates specific to the reduction mammaplasty subgroup [7]. Existing data and recommendations were largely confined to the hypermobile subtype, with minimal representation of other EDS subtypes.

## Conclusions

This systematic literature review and illustrative case emphasize the need for more studies regarding EDS and elective breast surgery. The illustrative case highlights that patients may present for surgery before receiving an EDS diagnosis, potentially increasing the risk of complications. It also identifies the unique role plastic surgeons may play in recognizing undiagnosed EDS and initiating referrals to rheumatology. While the illustrative case experienced postoperative complications, the systematic review uncovered a small number (3) of reported patients with macromastia and EDS who achieved symptomatic improvement following reduction mammaplasty with minimal complications. However, the limited number of studies underscores the need for further research. This review was intended to synthesize the available evidence on reduction mammaplasty in EDS, highlighting the importance of revisiting current practice patterns.
